# Demonstration of RNAi Yeast Insecticide Activity in Semi-Field Larvicide and Attractive Targeted Sugar Bait Trials Conducted on *Aedes* and *Culex* Mosquitoes

**DOI:** 10.3390/insects14120950

**Published:** 2023-12-15

**Authors:** Akilah T. M. Stewart, Keshava Mysore, Teresia M. Njoroge, Nikhella Winter, Rachel Shui Feng, Satish Singh, Lester D. James, Preeraya Singkhaimuk, Longhua Sun, Azad Mohammed, James D. Oxley, Craig Duckham, Alongkot Ponlawat, David W. Severson, Molly Duman-Scheel

**Affiliations:** 1 Department of Medical and Molecular Genetics, Indiana University School of Medicine, South Bend, IN 46617, USA; akilstew@iu.edu (A.T.M.S.); kmysore@iu.edu (K.M.); tenjorog@iu.edu (T.M.N.); lsun@nd.edu (L.S.); severson.1@nd.edu (D.W.S.); 2 Eck Institute for Global Health, The University of Notre Dame, Notre Dame, IN 46556, USA; 3 Department of Life Sciences, Faculty of Science & Technology, The University of the West Indies, St. Augustine Campus, St. Augustine, Trinidad and Tobago; nikhellaw@gmail.com (N.W.); shuifengr@gmail.com (R.S.F.); satishmsingh01@gmail.com (S.S.); lesterdjames@gmail.com (L.D.J.); azad.mohammed@sta.uwi.edu (A.M.); 4 Department of Entomology, US Army Medical Directorate–Armed Forces Research Institute of Medical Sciences (USAMD-AFRIMS), Bangkok 10400, Thailand; preerayas.ca@afrims.org (P.S.); alongkotp.fsn@afrims.org (A.P.); 5 Southwest Research Institute, San Antonio, TX 78238, USA; james.oxley@swri.org; 6 CD R&D Consultancy Services, Dorking RH4 2HF, UK; craig.duckham@cdrnd.co.uk; 7 Department of Biological Sciences, College of Science, The University of Notre Dame, Notre Dame, IN 46556, USA

**Keywords:** *Aedes aegypti*, *Aedes albopictus*, *Culex quinquefasciatus*, *Saccharomyces cerevisiae*, larvicide, insecticide, eco-friendly, mosquito control, *Shaker*, gene silencing

## Abstract

**Simple Summary:**

Mosquito-borne infectious diseases threaten millions of people worldwide, and it is critical that we identify new methods for controlling these insects. We recently developed a new class of mosquito insecticides that consists of yeasts which produce interfering RNA that is custom-designed to turn off mosquito genes yet does not hurt other organisms. Here, we test a newly optimized robust yeast strain which is suitable for industry-scale yeast production. Following heat inactivation and drying, the yeast was shipped to St. Augustine, Trinidad and Bangkok, Thailand, where it was evaluated in outdoor studies performed in tropical settings. The yeast effectively killed a variety of different types of mosquito larvae that died following yeast consumption. It could also be supplied to adult mosquitoes in a sugar bait solution, resulting in high levels of mosquito death. The yeast killed mosquitoes more quickly in outdoor experiments than in laboratory trials. These promising results indicate that yeast insecticides could one day be used globally as a new means of eco-friendly mosquito control and disease prevention.

**Abstract:**

Eco-friendly new mosquito control innovations are critical for the ongoing success of global mosquito control programs. In this study, Sh.463_56.10R, a robust RNA interference (RNAi) yeast insecticide strain that is suitable for scaled fermentation, was evaluated under semi-field conditions. Inactivated and dried Sh.463_56.10R yeast induced significant mortality of field strain *Aedes aegypti*, *Aedes albopictus*, and *Culex quinquefasciatus* larvae in semi-field larvicide trials conducted outdoors in St. Augustine, Trinidad, where 100% of the larvae were dead within 24 h. The yeast was also stably suspended in commercial bait and deployed as an active ingredient in miniature attractive targeted sugar bait (ATSB) station sachets. The yeast ATSB induced high levels of *Aedes* and *Culex* mosquito morbidity in semi-field trials conducted in Trinidad, West Indies, as well as in Bangkok, Thailand, in which the consumption of the yeast resulted in adult female mosquito death within 48 h, faster than what was observed in laboratory trials. These findings support the pursuit of large-scale field trials to further evaluate the Sh.463_56.10R insecticide, a member of a promising new class of species-specific RNAi insecticides that could help combat insecticide resistance and support effective mosquito control programs worldwide.

## 1. Introduction

Eco-friendly mosquito control innovations are vitally needed to address established and emerging arthropod-borne infectious diseases [1]. The successful deployment of RNAi pesticides, an innovative and relatively unexplored new eco-friendly mosquito control intervention [2], could significantly enhance mosquito control programs. High-throughput screens identified hundreds of insecticidal RNAi molecules, members of a new class of insecticides that could help combat resistance, which is an increasing concern for mosquito control programs [3]. A subset of these insecticides, which can be inexpensively produced and delivered to mosquitoes in the baker’s yeast *Saccharomyces cerevisiae*, kill *Aedes* (dengue, Zika, chikungunya, and yellow fever virus vectors), *Culex* (West Nile and lymphatic filariasis vector), and *Anopheles* (malaria vector) mosquitoes, but do not impact the survival of non-target organisms. Although these RNAi insecticides were initially identified in larvicide screens, several of the insecticides can also kill adult mosquitoes [3]. In this study, we examined the efficacy of the Sh.463_56.10R insecticide in semi-field deployment trials conducted on the Caribbean Island of Trinidad and in Bangkok, Thailand.

Our previous laboratory studies demonstrated that *Sh.463* interfering RNA designed to target a conserved site in mosquito *Shaker* genes, which encode voltage-gated potassium channels, results in the significant mortality of *Aedes, Anopheles*, and *Culex* spp. mosquitoes. Despite its impact on mosquitoes, Sh.463 yeast is not toxic to other non-target organisms [4]. Sh.463 and other RNAi yeasts can be incorporated into a heat-inactivated, dried, shelf-stable formulation that can be delivered to larvae in tablet form or to adult mosquitoes through the incorporation into sugar baits [3]. Here, we identify a yeast formulation that can be effectively shipped at room temperature to international destinations. Following international shipment, we evaluate the efficacy of the yeast when deployed as a larvicide in semi-field studies conducted on a rooftop laboratory in St. Augustine, Trinidad. For these studies, we selected the recently constructed second-generation Sh.463_56.10R yeast strain [5], a yeast which is more robust than the first-generation laboratory strains of *Saccharomyces cerevisiae* that were used in our previous studies [4], and which performs well in scaled yeast fermentations [5]. The Sh.463_56.10R yeast strain was generated with the RNA-guided Cas-CLOVER system, which was used in combination with Piggybac transposase to generate a *Saccharomyces cerevisiae* strain bearing multiple integrated copies of the *Sh.463* shRNA insecticide expression cassette. This yeast produces high levels of the *Sh.463* shRNA insecticide, which was placed under the control of a constitutively active promoter [5].

In addition to pursuing semi-field larvicide trials with Sh.463_56.10R yeast, it was evaluated in adult female mosquitoes by testing it as the active ingredient in ATSBs. ATSBs, a new paradigm for vector control, capitalize on the innate sugar feeding behavior of female and male mosquitoes that consume sugar bait spiked with an insecticide [6]. In field trials conducted in Mali, ATSBs significantly reduced the density of *Anopheles* malaria vector mosquitoes [7]. ATSB bait stations that deploy the broad-based insecticide dinotefuran are currently being assessed in large-scale clinical trials as an *Anopheles* mosquito control tool for malaria prevention in Africa [8]. The success of these field trials will advance ATSBs toward consideration as a new class of World Health Organization (WHO)-registered interventions for mosquito control. This could eventually enable the registry of second-generation ATSB insecticides, potentially including mosquito-specific yeast RNAi insecticides that may enhance existing ATSB technology. While sugar baits enable a much more targeted delivery of insecticides, resistance is nevertheless still of concern, and new classes of insecticides such as RNAi yeast are therefore of high interest. Moreover, second-generation insecticides that are specific to mosquitoes, such as RNAi insecticides, are of particular interest. In this study, we use a miniature bait station system designed to mimic that which is being assessed in the ongoing African malaria vector trials [8] to evaluate the impact of Sh.463_56.10R RNAi yeast ATSBs on *Aedes* and *Culex* mosquitoes in semi-field trials conducted in Trinidad and Thailand, two countries with tropical conditions, high abundances of mosquitoes, and frequent mosquito-borne disease transmission, and which would greatly benefit from new mosquito control interventions. Although the current clinical trials in Africa [8] are focusing on malaria vector mosquitoes, here, we focus on the analysis of *Aedes* and *Culex* mosquitoes.

## 2. Materials and Methods

### 2.1. Animal Rearing

*St. Augustine*, *Trinidad*: The *A. aegypti*, *A. albopictus*, and *C. quinquefasciatus* mosquitoes used in this investigation were collected locally and used to establish mosquito strains, which were reared in the University of the West Indies (UWI) insectary. This insectary is maintained at local ambient temperature and humidity conditions. For blood feeding, sheep’s blood (School of Veterinary Medicine, Faculty of Medical Sciences, Mt. Hope) was supplied to adult females through a parafilm membrane. The mosquitoes used in this semi-field investigation were roughly fifth-generation mosquitos and beyond.

*Bangkok*, *Thailand*: The *A. aegypti* (Phitsanulok field strain) larvae were collected in March 2023 from human-made containers at a village (16°34′00.8″ N 100°41′40.9″ E) located in Noen Maprang District, Phitsanulok Province, Thailand. To produce F1 progeny for the semi-field experiment, they were maintained in the insectary at the Department of Entomology, AFRIMS, at 28 ± 2 °C, 80% relative humidity, and under a light regime of 12 light/12 h dark (L:D). Human blood meals were provided using an artificial membrane feeding technique. To ensure sugar starvation, 10% sucrose was removed from the cages 24 h before the yeast feeding experiment.

### 2.2. Yeast Culturing and Preparation

The genotypes of the yeast strains used in this investigation are as follows: Sh.463_56.10R genotype: *MATa*, *PiggyBac* (*leu2d/P_TDH3_-shRNA_463-T_CYC1_*, *P_TDH3_-shRNA_463-T_CYC1_*, *P_TDH3_-shRNA_463-T_CYC1_*), *CEN/ARS* (*URA3/SPBase-Sc-CO*); Control_347.1R (referred to simply as Control herein) genotype: *MATa, PiggyBac* (*LEU2/P_TDH3_-shRNA_Ctrl-T_CYC1_*), *2um* (*URA3/SPBase_Sc-CO*), *PiggyBac* (*HIS3/P_TDH3_-shRNA_Ctrl-T_CYC1_*), *CEN/ARS* (*URA3/SPBase_Sc-CO*) *PiggyBac* (*trp1d/P_TDH3_-shRNA_Ctrl-T_CYC1_*), *CEN/ARS* (*URA3/SPBase_Sc-CO*); note that this strain expresses a hairpin with no known target in mosquitoes. The construction of these yeast strains was described previously [5].

### 2.3. Yeast Preparation

The yeasts were cultured, heat-killed, and pelleted as described [9]. The pelleted yeast was lyophilized with 0.1% benzoic acid (preservative) using a Labconco FreeZone 6 L Console Freeze Dryer (Labconco, Kansas City, MO, USA).

### 2.4. Yeast Shipment

Yeast was shipped from Indiana University School of Medicine to St. Augustine, Trinidad or to Thailand at ambient temperatures via standard Federal Express procedures. On average, shipments arrived within one week. Prior to shipment and upon arrival at the final destination, yeast was stored at −20 °C until use.

### 2.5. Semi-Field Larvicide Trials

The semi-field larvicide trials were performed at an outdoor rooftop laboratory at UWI, St. Augustine, Trinidad in summer 2023. These trials were performed as previously described [9] on locally obtained mosquitoes while conforming to the WHO larvicide testing guidelines [10]. Nine replicates, each with 20 first instar (L1) larvae, were evaluated for each treatment. A total of 20 larvae were placed in 3.7 L of distilled water in a 7.5 L ovitrap container that was treated with 40 mg of heat-inactivated dried yeast; the container was covered with mesh and placed in a screened SansBug 1-Person Free-Standing Pop-Up Mosquito-Net tent (Hakuna Matata Tents, Markham, ON, Canada) situated underneath a canopy. The mesh on the containers and the tent was used to prevent mosquito escape and the entrance of macrobiota into the trial site. During the testing period, temperatures ranged from 23.6 °C to 38 °C. Larvicide trial data were analyzed using Student’s *t*-test.

### 2.6. RNAi Yeast ATSB Sedimentation Studies

An amount of 1 mL of samples was taken from 35 mL suspensions of 0.4 mg/mL lyophilized inactive RNAi yeast suspended in BaitStab^TM^ lacking dinotefuran (Westham Co., Tel Aviv, Israel) [11], which had been prepared through hand mixing with a spatula. The bait contained uranine, which fluoresces, allowing for the confirmation of mosquito feeding. The yeast-BaitStab^TM^ suspension was evaluated with a Lumisizer (Berlin, Germany), which is used to quantify creaming and settling. In these studies, which were performed at the Southwest Research Institute in San Antonio, Texas, the yeast ATSB suspension was heated to 75.0 °C and transferred to Lumisizer 1 mm pathlength cuvettes with a volume of approximately 0.2 mL. The samples were run under the following conditions: 865 nm wavelength, a 104.53 mm meniscus, 300 profiles at 75 s intervals at a speed of 4000 rpm, a light factor of 1.00, and a temperature of 60 °C.

### 2.7. Semi-Field Yeast ATSB Trials

*St. Augustine*, *Trinidad*: Semi-field ATSB trials were performed at the UWI, St. Augustine rooftop laboratory in summer 2023. An amount of 8 mg of lyophilized yeast (Sh.463_56.10R or control) containing 0.1% benzoic acid was combined with 200 μL of BaitStab^TM^ lacking dinotefuran (provided by Westham Co.), and the suspension was placed on a 1.3 cm square of thick plastic sheet and covered with Westham membrane (Westham Co.). The membrane was sealed with a heat sealer to create a miniature membrane feeder (sachet). A total of 25 adult female mosquitoes were placed in a 3.75 L (30 cm × 30 cm × 20 cm) insect cage that was situated inside the tents used in the larvicide trials (see above). A sachet bait station was hung along the top of one side wall in a 3.75 L experimental cage with the Westham Co. membrane facing inward; this facilitated feeding, which was permitted throughout the experimental period that commenced immediately following a 24 h period of sugar starvation. Water was supplied with a wetted cotton ball during each trial. Female engorgement, confirmed through the visualization of fluorescent dye in mosquito abdomens, verified feeding. Each experiment was conducted in triplicate, monitoring daily survival rates over the next six days. Temperatures ranged from 23.6 °C to 38 °C. Relative humidity ranged from 32.0 to 100.0%. Survival rates were compared using ANOVA with Tukey’s post hoc test.

*Thailand*: The methods in Thailand were comparable to those applied in Trinidad, except the mosquitoes were housed in a 0.375 L cage. An amount of 80 mg of yeast was diluted in 200 μL of BaitStab^TM^. During the trials, the relative humidity ranged from 52.5 to 71.8%, and temperatures ranged from 30.1 to 33.7 °C.

## 3. Results and Discussion

### 3.1. Semi-Field Larvicide Activity of Sh.463_56.10R Yeast

**Figure 1:**
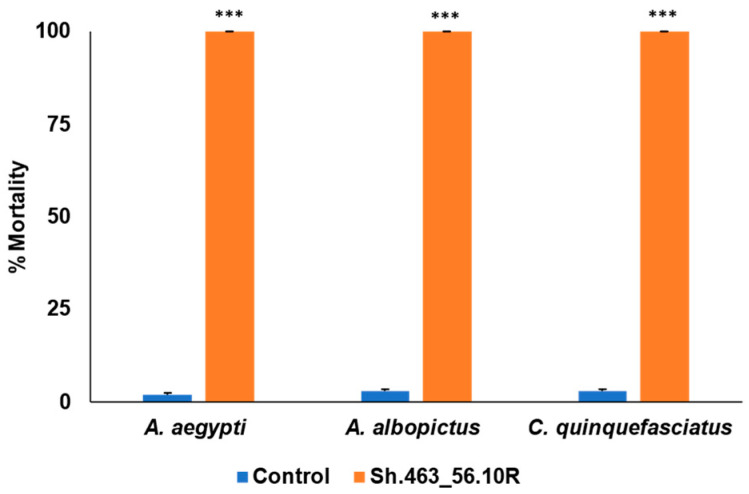
Semi-field evaluation of Sh.463_56.10R yeast larvicide activity in St. Augustine, Trinidad. Sh.463_56.10R yeast retained significant larvicidal activity following shipment to Trinidad, where semi-field trials were conducted using field strains of larvae of the indicated species. Mean results from eight replicate trials, each with 20 larvae per treatment, are shown. Error bars denote SEM. *** = *p* < 0.001 with respect to control yeast treatments.

Sh.463_56.10R yeast was heat-inactivated and lyophilized with benzoic acid as a preservative, and then shipped to St. Augustine, Trinidad, where it was evaluated in outdoor semi-field larvicide trials. These trials, which were conducted in a rooftop laboratory within a canopy tent, were conducted using local field strains of *A. aegypti*, *A. albopictus*, or *C. quinquefasciatus* mosquitoes, all of which are abundant in Trinidad. Although nearly all larvae treated with a control RNAi strain of yeast survived, the Sh.463_56.10R yeast treatments resulted in 100% larval mortality in all three species of mosquitoes (Figure 1). These mortality levels are higher than those reported in laboratory trials, in which mortality rates of ~90% were observed in conjunction with containers bearing 20 larvae treated with either first-generation [4] or second-generation RNAi yeast insecticides targeting the *Sh* gene [5] or other vital mosquito genes. These results demonstrated a high level of efficacy for the yeast which had been shipped overseas at room temperature, and which took approximately one week on average to reach its tropical destination.

**Figure 2:**
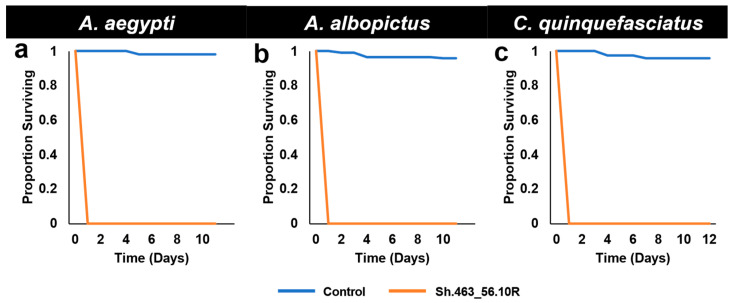
Survival following semi-field larvicide treatments. Although control RNAi yeast-treated larvae of the indicated species survived development under semi-field trial conditions in St. Augustine, Trinidad, *A. aegypti* (**a**), *A. albopictus* (**b**), and *C. quinquefasciatus* (**c**) larvae that consumed Sh.463_56.10R yeast died within one day following treatment. Results were compiled from the trial data shown in Figure 1.

In addition to the high mortality levels observed in Trinidad, in these semi-field trials, the yeast treatments led to the death of the first instar larvae on the first day of treatment (Figure 2). The rate of death induced by the yeast in these studies, which were completed in a high-temperature environment, was faster than those achieved under laboratory insectary conditions. In the insectary trials, the yeast strains examined to date, including those completed with Sh.463_56.10R yeast [5], induced death in the late third or fourth instar following continuous yeast consumption throughout the larval period [4,12,13]. Likewise, semi-field trials conducted in a more temperate environment in Indiana yielded results that are more similar to those obtained in the laboratory, with death occurring in the late third or early fourth instar for all strains tested to date [12,13], including the original Sh.463 yeast strain [4]. These results suggest that temperature is likely a critical determinant of RNAi larvicide activity.

The faster rates of death observed outdoors, which could potentially be the result of additional stress on the larvae in a high-temperature outdoor environment or shifting between daytime and nighttime temperatures, bodes well for the potential implementation of RNAi yeast larvicides as a new mosquito control intervention in the tropics. RNAi has been reported to work more efficiently at higher temperatures in *C. elegans* [14]. Likewise, an increased temperature was reported to lead to the faster manifestation of RNAi phenotypes in the flatworm *Macrostomum lignano* [15]. In insects, research has demonstrated that RNAi is impaired in *A. aegypti* reared at cooler temperatures [16]. Similarly, in *D. melanogaster,* RNAi was also found to be sensitive to temperature, with decreased efficiency at lower temperatures and increased efficiency at higher temperatures; however, at lower temperatures, the RNAi rates became more dependent on the availability of interfering RNA [17]. These studies suggest that tropical temperatures may increase the RNAi rates, leading to faster rates of mortality following Sh.463_56.10R larvicide treatments. At lower temperatures, it may be useful to increase the pesticide concentration, a phenomenon that could be assessed in larger field trials conducted in more temperate climates.

### 3.2. Development of a Stable Yeast RNAi ATSB Suspension

The next goal was to determine if the RNAi yeast could be incorporated into ATSBs. To achieve this, the dried inactivated lyophilized yeast containing benzoic acid as a preservative was prepared in BaitStab^TM^ (Westham Co.), the attractive sugar bait (ASB) which is presently being used to deliver dinotefuran to mosquitoes in the ongoing field trials in Africa [8]. BaitStab^TM^ contains date syrup and brown sugar that promote mosquito attraction and gustation responses. Following the hand mixing of dried yeast and BaitStab^TM^, the sedimentation properties of the yeast suspension were examined.

**Figure 3:**
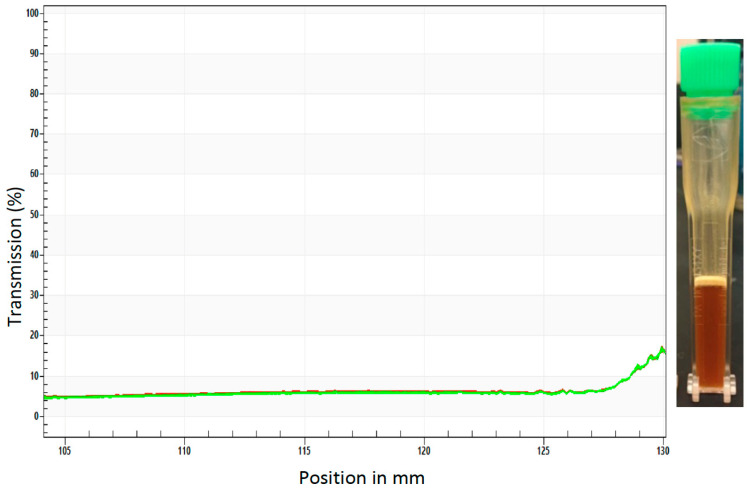
Sedimentation analysis of the yeast ATSB suspension. Uniform transmittance was detected throughout the profile of the cuvette (shown at right) during sedimentation analysis of the yeast ATSB suspension at 4000 rpm for six hours (300 profiles, 75 s intervals, light factor = 1.0, temperature = 60 °C). Each of the 300 profiles is included in the plot with a color transition from red to green for each plotted line.

During a sedimentation analysis, no changes were observed when the sample was spun at 4000 rpm over 40 min at room temperature, 4000 rpm over 40 min at 60 °C, or for 6+ h at 60 °C (Figure 3). Although some creaming was observed after 6+ h under these conditions (Figure 3), no evidence of settling was detected. No change in transmittance, which was uniform throughout the profile of the cuvette, was observed, indicating that there is no change in the optical composition/density (no settling). These results suggest that upon the thorough mixing of the dried yeast into the BaitStab^TM^, the ATSB suspension is stable. These results also indicated that the suspension may be suitable for outdoor deployment. To further investigate this, Sh.463_56.10R yeast was cultured, heat-inactivated, dried, and then shipped to Trinidad, where it was suspended in BaitStab^TM^ and evaluated in outdoor semi-field trials.

### 3.3. Semi-Field Trial Confirmation of Yeast RNAi ATSB Activity

**Figure 4:**
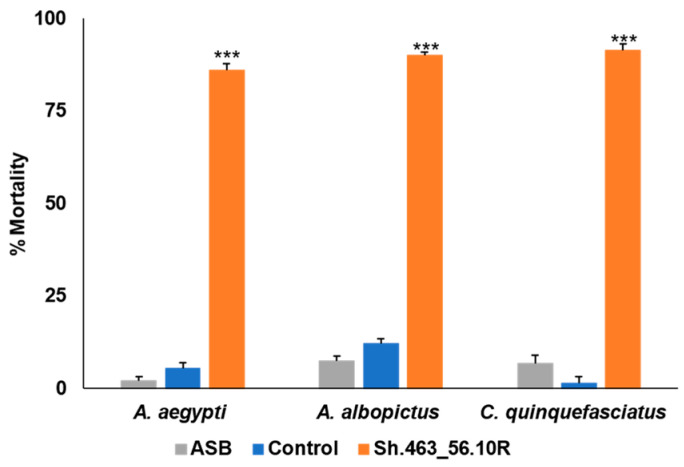
Semi-field confirmation of Sh.463_56.10R yeast ATSB activity in St. Augustine, Trinidad. Sh.463_56.10R yeast retained significant adulticidal activity following shipment to Trinidad, where it was delivered as an ATSB to adult female mosquitoes of the indicated field strains. Mean results from three replicate trials, each conducted with 25 adult females of the indicated species, are shown. Error bars denote SEM. *** = *p* < 0.001 with respect to either ASB or control yeast treatments.

The RNAi yeast–BaitStab^TM^ mixture was prepared and delivered to adult *Aedes*, *Culex*, and *Anopheles* mosquitoes using a miniature bait station sachet designed to mimic that which is being evaluated in the ongoing ATSB trials in Africa [8]. The stations were evaluated in outdoor semi-field trials conducted in St. Augustine, Trinidad. Morbidity induced by the Sh.463_56.10R yeast ATSB was examined in adult female *A. aegypti*, *A. albopictus*, and *C. quinquefasciatus* mosquitoes. The ATSB was marked with fluorescent dye, which was used to confirm that the mosquitoes had consumed it. Sh.463_56.10R yeast ATSB induced significant (*p* < 0.001) 90–100% morbidity in the semi-field trials conducted on each of these species of mosquitoes (Figure 4).

**Figure 5:**
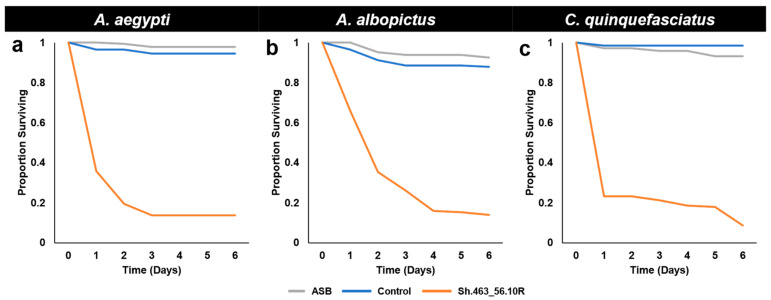
Survival following ATSB consumption in semi-field trials. Although adult females of the indicated species survived for six days following the consumption of sugar bait (ASB) or control yeast, the majority of *A. aegypti* (**a**), *A. albopictus* (**b**), and *C. quinquefasciatus* (**c**) adult female mosquitoes died within 24–48 h following the consumption of Sh.463_56.10R yeast. Results were compiled from the trial data shown in Figure 4.

As observed in the larvicide trials, the extensive morbidity observed in the semi-field ATSB trials (Figure 4) typically occurred within 24–48 h after the initiation of the treatment (Figure 5). This is faster than the death rates observed in the laboratory, where death typically occurred within 3–6 days following the treatment with Sh.463_56.10R yeast, even when it was prepared in a 5-fold more concentrated suspension [5]. The locomotor defects that were previously observed following the silencing of the mosquito *Shaker* gene with the first-generation Sh.463_56.10R yeast strain [4] were also observed in these outdoor trials conducted with Sh.463_56.10R yeast. This phenotype likely contributed to the faster rate of death observed outdoors, as the mosquitoes could not maintain adequate hydration levels and became dehydrated quickly in the tropical outdoor climate. Moreover, as discussed with respect to the results of the larvicide trials (Figure 2), RNAi may be more efficient at the higher temperatures observed in outdoor tropical environments.

**Figure 6:**
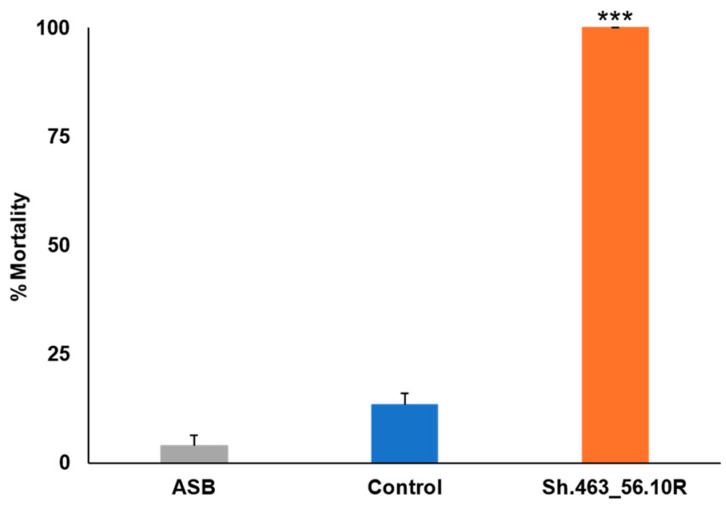
Semi-field confirmation of Sh.463_56.10R yeast ATSB activity in Bangkok, Thailand. Sh.463_56.10R yeast induced significant adult morbidity during semi-field trials conducted on field strain adult female *A. aegypti* mosquitoes. Mean results from six replicate trials, each conducted with 25 adult females, are shown. Error bars denote SEM. *** = *p* < 0.001 with respect to either ASB or control yeast treatments.

Comparable results were observed in the ATSB trials conducted in Bangkok, Thailand, where Sh.463_56.10R yeast that had been shipped from Indiana to Bangkok induced 100% morbidity in *A. aegypti* adult female mosquitoes, while the negative control group showed only 1% mortality (Figure 6). The results revealed a significant difference in mortality rates between the experimental group treated with Sh.463_56.10R yeast and the negative control group (*p* < 0.001). As observed in Trinidad, mosquitoes treated with Sh.463_56.10R yeast died within 24–48 h of treatment. Combined, these findings suggest that the Sh.463_56.10R yeast adulticide can induce high levels of *Aedes* and *Culex* mosquito mortality, making it a potential innovative biorational method for mosquito control.

### 3.4. Conclusions and Future Work

In conclusion, the results of these assays indicate that the yeast interfering RNA larvicide induced significant larval death following international shipment at room temperature, as ascertained in semi-field trials in which 100% of the larvae died within one day (Figure 1 and Figure 2). These results support the large-scale field testing of Sh.463_56.10R yeast as a larvicide. Given that the Sh.463_56.10R [5] yeast strain is suitable for scaled fermentations, generating sufficient material for large field trials is now feasible. These advancements are timely, given the increasing worldwide threats posed by arboviral illnesses [18,19], combined with recent concerns for the spread of *Anopheles stephensi* mosquitoes, malaria vector mosquitoes that are susceptible to larvicides, into Africa [20]. Moreover, this same dried yeast was also successfully suspended and delivered to adults in BaitStab^TM^ (Figure 3), in which it induced high levels of adult *Aedes* and *Culex* mosquito morbidity in semi-field trials conducted in the Caribbean (Figure 4 and Figure 5) and in Southeast Asia (Figure 6).

Bacterial systems for interfering RNA expression and delivery have also been explored. For example, *Escherichia coli*, which was initially used as an interfering RNA delivery system in *Caenorhabditis elegans* [21], has also been used successfully for the delivery of RNA to mosquito larvae [22] and adults [23]. Like yeast, bacterial RNAi systems also generate high levels of silencing in mosquitoes [22,23]. However, as previously discussed [3], it is anticipated that stakeholders may prefer the use of baker’s yeast rather than bacteria near their homes. Moreover, yeast is highly attractive to mosquitoes and may therefore promote increased mosquito attraction and feeding [3]. Of course, it will be important to assess whether the attractiveness of the yeast ATSB might lure other insect pests to the bait stations. If this occurs, the membrane used to create the bait stations deployed in the ongoing African field trials [8] was designed to allow for penetration only by insects with a proboscis. The selectivity of the membrane combined with the narrow range of the yeast pesticide should allow for yeast RNAi ATSB bait stations to selectively kill mosquitoes.

Dinotefuran-based ATSBs drastically reduced the density of *Anopheles* malaria vector mosquitoes in large-scale field trials in Mali [7]. A successful outcome for the ongoing dinotefuran-based ATSB bait station trials that are assessing the impact of this intervention on malaria [8] could potentially advance WHO approval for ATSBs as a new class of mosquito control interventions. This might, in turn, open the potential for the registry of second-generation ATSBs that include RNAi yeast as an active ingredient. RNAi yeast ATSBs could enhance existing ATSB technology as a mosquito-specific next-generation ATSB, the use of which could help combat insecticide resistance, a growing problem which must be addressed by the development of new interventions [24,25,26]. The specificity of the *Sh.463* shRNA insecticide for mosquitoes, as illustrated by the observed lack of impact of this insecticide on other insects [4], makes it an eco-friendly alternative to broad-based insecticides. It would be interesting to pursue similar trials to assess the effect of RNAi yeast ATSBs on *Anopheles* mosquitoes, in which the target site of Sh.463 is conserved [4]. The results of this investigation suggest that larger field trials should also be performed to assess the impact of RNAi yeast ATSBs on *Aedes* and *Culex* mosquitoes, which were highly susceptible to this intervention.

The pursuit of larger field trials will, of course, require permit acquisition in each country in which the trials will be conducted. Furthermore, the registry of the RNAi yeast insecticides as a new active ingredient, which we hope to pursue with the United States (U.S.) Environmental Protective Agency (EPA), will require field testing at U.S. field sites. When considering the risk of using the yeast RNAi insecticides in such trials, it is important to remember that the yeast, which is heat-inactivated prior to deployment, is a dead microbial. Although the use of the yeast will therefore not require the release of live genetically modified organisms, the EPA has actually already registered one live genetically modified RNAi-based agricultural intervention: transgenic corn expressing double-stranded RNA comprising a *DvSnj7* inverted repeat sequence derived from the Western corn rootworm *Diabrotica virgifera virgifera* [27]. This registry decision paved the way for a second insecticidal RNAi intervention that the EPA is presently considering [28], as well as a future registry application for a yeast RNAi mosquito control intervention. In addition to data demonstrating the lack of *Sh.463* hairpin impacts on the survival of non-target arthropods, the submission of a yeast RNAi registry application will likely require the pursuit of further toxicological studies to be specified by the EPA in pre-registry discussions. The extent of these studies will be dependent on the intended uses of the yeast. For example, if the yeast is to be used as a larvicide for the treatment of open bodies of water rather than small water containers, then the potential for yeast-treated water to enter larger water systems bearing other invertebrates or fish may need to be considered. If the yeast larvicides are to be used to treat drinking water stored in containers, a more thorough demonstration of their safety in humans will likely be required [29,30].

Beyond pursuing work in additional species of mosquitoes, it would also be interesting to develop RNAi yeast pesticides for the control of other insect species. RNAi yeast strains were successfully evaluated in *Drosophila suzukii* [31], commonly known as spotted wing *Drosophila* (SWD), an invasive agricultural pest that is wreaking havoc on the fruit industry [32,33]. Given that SWD will drink sugar baits and are attracted to yeast-baited traps [34], it would be useful to evaluate whether yeast RNAi ATSBs could be designed to specifically target this crop pest. Moreover, it would be useful to design and evaluate RNAi yeasts targeting other insect pests, for example, ants and cockroaches, for which there is considerable interest in the development of RNAi pesticides [35,36]. The potential use of yeast RNAi for the control of Lepidopteran crop pests, for which highly variable responses to RNAi treatments have been observed in conjunction with other RNA delivery systems [37], could be valuable. In addition to such efficacy studies, it will be important to pursue active community engagement to ascertain public feelings regarding RNAi pesticide technology. These studies would serve to provide stakeholders with clear, balanced, and objective information regarding yeast RNAi pesticide technology and its intended uses, and to discuss any relevant risks, as well as the costs and benefits of the new intervention [38]. Such engagement studies, which are now becoming a standard component of developing new mosquito control interventions [39,40], should also be extended to agricultural stakeholders when new RNAi pesticides targeting crop pests are being developed. Our successful development and evaluation of the Sh.463 yeast RNAi pesticide suggests that further insecticide development and stakeholder engagement is merited.

## Data Availability

All data are contained within the article.
